# Celastrol induces apoptosis and autophagy via the ROS/JNK signaling pathway in human osteosarcoma cells: an *in vitro* and *in vivo* study

**DOI:** 10.1038/cddis.2014.543

**Published:** 2015-01-22

**Authors:** H-Y Li, J Zhang, L-L Sun, B-H Li, H-L Gao, T Xie, N Zhang, Z-M Ye

**Affiliations:** 1 Department of Orthopedics, Second Affiliated Hospital, School of Medicine, Zhejiang University, Hangzhou, China

## Abstract

Osteosarcoma is the most common primary malignant tumor of bone, the long-term survival of which has stagnated in the past several decades. Celastrol, a triterpene from traditional Chinese medicine, has been proved to possess potent anti-tumor effect on various cancers. However, the effect of celastrol on human osteosarcoma and the underlying mechanisms remains to be elucidated. We reported here that celastrol could inhibit cell proliferation by causing G2/M phase arrest. Exposure to celastrol resulted in the activation of caspase-3, -8, and -9, indicating that celastrol induced apoptosis through both extrinsic and intrinsic pathways. Autophagy occurred in celastrol-treated cells as evidenced by formation of autophagosome and accumulation of LC3B-II. The celastrol-induced cell death was remarkably restored by the combination of autophagy and apoptosis inhibitors. Furthermore, inhibition of apoptosis enhanced autophagy while suppression of autophagy diminished apoptosis. Celastrol also induced JNK activation and ROS generation. The JNK inhibitor significantly attenuated celastrol-triggered apoptosis and autophagy while ROS scavenger could completely reverse them. The ROS scavenger also prevented G2/M phase arrest and phosphorylation of JNK. Importantly, we found that celastrol had the similar effects on primary osteosarcoma cells. Finally, *in vivo*, celastrol suppressed tumor growth in the mouse xenograft model. Taken together, our results revealed that celastrol caused G2/M phase arrest, induced apoptosis and autophagy via the ROS/JNK signaling pathway in human osteosarcoma cells. Celastrol is therefore a promising candidate for development of antitumor drugs targeting osteosarcoma.

Osteosarcoma is the most common primary malignant tumor of bone, occurring predominantly in children and adolescents with a very high propensity for local invasion and early systemic metastases.^1^ The 5-year survival of patients with localized osteosarcoma has improved to 60% due to the multi-agent, dose-intensive chemotherapy in conjunction with gradually improved surgical techniques, but has remained largely unchanged during the last three decades.^2^ At the same time, the high-dose use of chemotherapeutic drugs is limited due to their systemic toxicity. Therefore, the development of novel therapies for the management of osteosarcoma is especially urgent.

Celastrol, a triterpenoid isolated from the traditional Chinese medicine ‘Thunder of God Vine', has been used in the treatment of autoimmune and neurodegenerative diseases.^3, 4, 5^ Recently, celastrol has attracted great attention for its potent anticancer effects and its diverse molecular targets involved in tumorigenesis have been reported.^6, 7, 8, 9, 10, 11, 12^ Although these molecular targets have positive correlations with inhibition of tumors, it is not clear which, if any, is the direct target or the principal mediator. Meanwhile, whether celastrol suppresses the growth of human osteosarcoma has never been investigated before.

Cell cycle deregulation is a hallmark of tumor cells and defective checkpoint function results in genetic modifications that contribute to tumorigenesis.^13^ The G2/M checkpoint is one of the conspicuous targets for anticancer drugs. The cyclin B1/ Cdc2 complex, which plays a key role in promoting the G2/M phase transition, is regulated by a range of proteins, including Cdc2, Cdc25C, Chk1/2 and p21.^14, 15, 16^

Cell death could be classified into apoptosis, autophagy, necrosis, cornification and tentative definitions of atypical cell death modalities.^17^ Apoptosis, as the type-I programmed cell death (PCD), plays a key role in chemotherapies against a variety of cancers.^18^ Autophagy is regarded as type-II PCD and the caspase-independent cell death pathway.^19^ In some cellular settings, autophagy serves as a cell survival pathway, suppressing apoptosis, and, in others, it can lead to cell death itself, either in collaboration with apoptosis or as a back-up mechanism when the former is defective.^20^ Whether celastrol can induce apoptosis or autophagy, what roles do they have and the interplay between each other in celastrol-induced cell death of osteosarcoma remain to be determined.

Reactive oxygen species (ROS), active forms of oxygen, generate as by-products from cellular metabolism.^21^ A moderate increase in ROS can promote cell proliferation and differentiation, whereas excessive amounts of ROS can interfere with cellular signaling pathways by causing oxidative damage to lipids, proteins and DNA.^22, 23, 24^ Interestingly, accumulating evidence suggests that cancer cells are under increased oxidative stress, and therefore more vulnerable to damage by further ROS insults induced by exogenous agents.^25^ Furthermore, ROS could affect various signaling pathways such as MAPK signal transduction cascades.^26, 27^ As a stress-activated protein kinase (SAPK) of the MAPK family, JNK plays a pivotal role in many cellular events, including apoptosis and autophagy.^28, 29^ Accordingly, targeted inhibition of related signaling pathways, particularly the ROS/JNK signaling, may be effective in the treatment of human cancers.

In the present study, we elucidated the inhibitory effect of celastrol on osteosarcoma cell lines and primary cells *in vitro* and *in vivo*. We further explored the molecular mechanisms, that is, induction of G2/M phase arrest, apoptosis and autophagy mediated by the ROS/JNK signaling pathway.

## Results

### Celastrol inhibits the proliferation of osteosarcoma and less cytotoxic to fibroblasts

**Figure 1:**
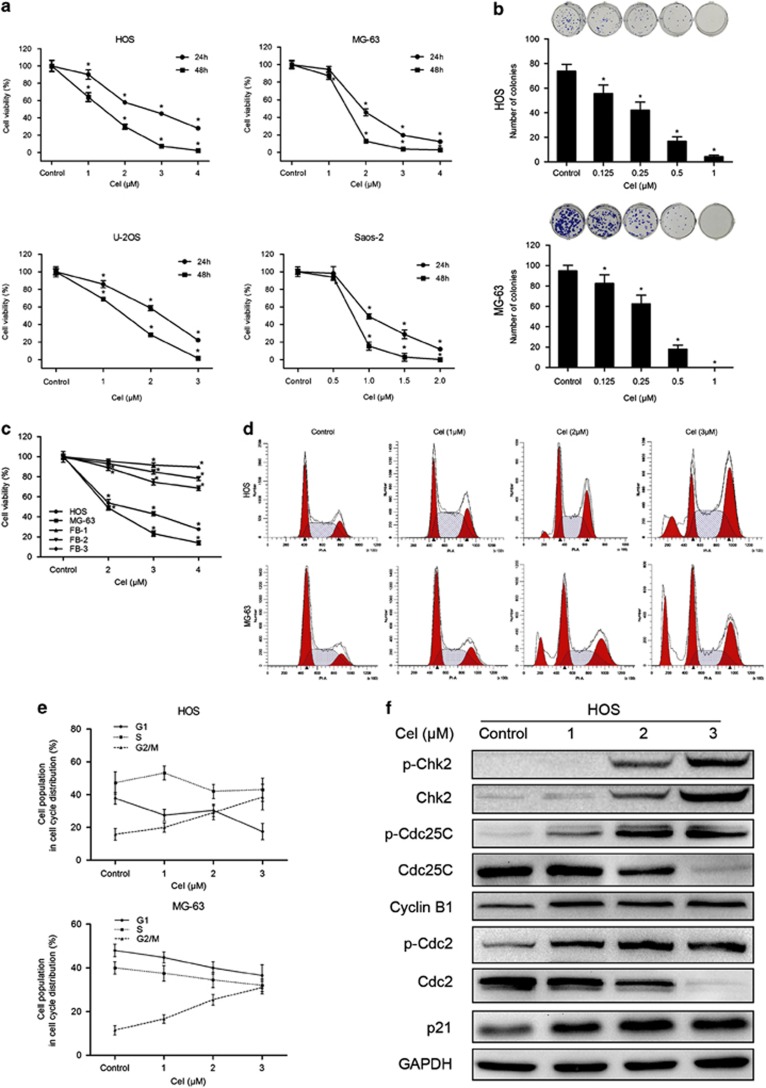
Celastrol inhibits cell proliferation and induces G2/M phase arrest in human osteosarcoma cells. (**a**) The anti-proliferative effect of celastrol on four osteosarcoma cell lines was determined by MTS. Cells were treated with various concentrations of celastrol for 24 and 48 h. Control group contained 0.1% DMSO. Data represented the mean of five replicates. Each performed in triplicate. (**b**) Colony formation assay of HOS and MG-63 cells with control or celastrol. (**c**) Comparison of the effect of celastrol on three normal human primary skin fibroblast samples with that on osteosarcoma cells for 24 h. (**d**, **e**) Celastrol induced G2/M phase arrest. Cells were treated with control or celastrol for 24 h and analyzed by flow cytometry. The percentage of cell cycle distribution was presented as the mean±S.D. from three independent experiments. (**f**) HOS cells were treated with celastrol for 24 h. The expressions of cell cycle-regulated proteins were measured by western blot. *P<0.05, significantly different compared with control

To investigate the effect of celastrol on growth, HOS, MG-63, U-2OS and Saos-2 cells were exposed to various concentrations for 24 and 48 h (Figure 1a). The IC_50_ values of celastrol for 24 h were 2.55 *μ*M for HOS, 1.97 *μ*M for MG-63, 2.11 *μ*M for U-2OS and 1.05 *μ*M for Saos-2 cells. Colony formation assay showed fewer colonies formed after celastrol treatment (Figure 1b). Interestingly, human fibroblasts showed strong resistance to celastrol, the IC_50_ values for which were 22.11, 8.33 and 6.32 *μ*M, respectively (Figure 1c). These results show that celastrol inhibits the proliferation of osteosarcoma in a dose- and time-dependent manner and less cytotoxic against normal cells.

### Celastrol induces G2/M phase arrest by regulating cell cycle-regulated proteins

To determine whether celastrol inhibits cell proliferation by inducing cell cycle arrest, we examined the distribution of cell cycle in cells treated with celastrol. As shown in Figures 1d and e, celastrol led to the accumulation of cells in G2/M phase and a corresponding decrease in G0/G1 and S phases in both HOS and MG-63 cells. To elucidate the mechanisms, we measured the expressions of cell cycle-regulated proteins. Celastrol upregulated the expressions of phospho-Chk2, Chk2, phospho-Cdc25C, phospho-Cdc2, p21, cyclin B1 and downregulated the levels of Cdc25C and Cdc2 (Figure 1f). All the data suggest that celastrol induces G2/M phase arrest by altering the key molecules of G2/M cell cycle regulator markers.

### Celastrol induces mitochondria-mediated apoptosis

**Figure 2:**
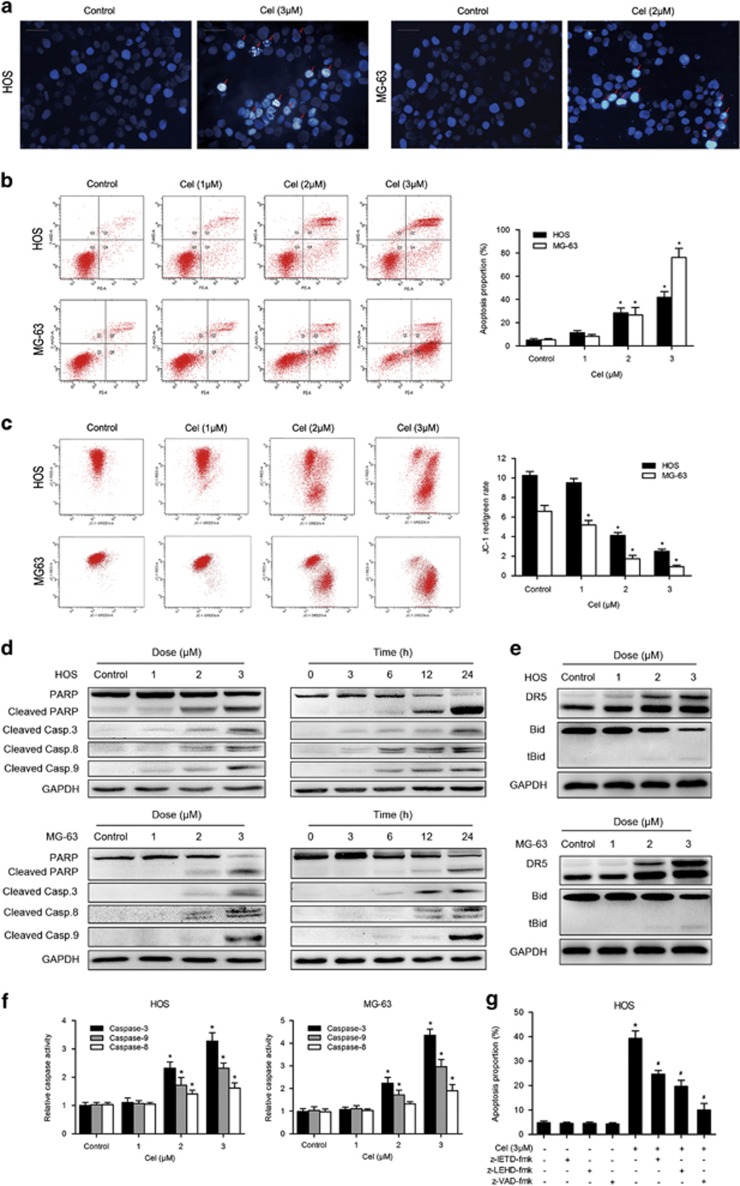
Evidence that celastrol induces apoptosis in osteosarcoma cells. (**a**) Apoptotic morphological changes were evaluated by fluorescent microscopy using Hoechst 33258 staining. Arrows indicate chromatin condensation and DNA fragmentation. Bar: 50 *μ*m. (**b**) HOS and MG-63 cells treated with celastrol were stained with annexin V-PE/7-AAD and analyzed by flow cytometry. The chart illustrates apoptosis proportion from three separate experiments. (**c**) The mitochondrial membrane potential was measured with JC-1 fluorescent probe and assessed by flow cytometry. The chart illustrates changes of JC-1 red/green rate from three independent experiments. (**d**, **e**) Cells were treated with various concentrations of celastrol for 24 h or incubated with celastrol (3 *μ*M) for different hours. The expressions of cleaved PARP, caspase-3, -8, -9, DR5 and Bid were determined by western blot. (**f**) Caspase activity assay of cells treated with various concentrations of celastrol for 24 h. (**g**) HOS cells were incubated with or without celastrol for 24 h after 2 h pre-treatment with caspase inhibitors, z-IETD-fmk (10 *μ*M), z-LEHD-fmk (40 *μ*M) or z-VAD-fmk (20 *μ*M). Then cells were stained with annexin V-PE/7-AAD and analyzed by flow cytometry. Results are expressed as the mean±S.D. from three independent experiments. *P<0.05 *versus* control, ^#^*P*<0.05 *versus* celastrol treatment

**Figure 3:**
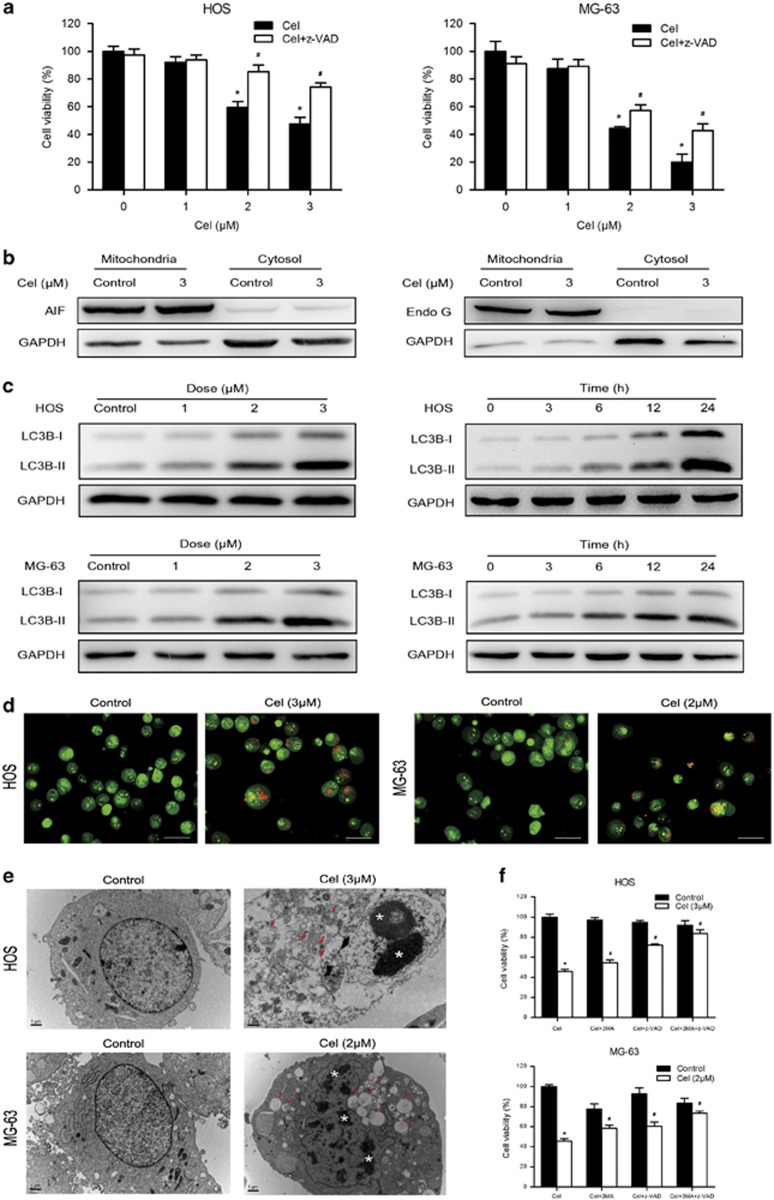
Celastrol induces autophagy, which contributes to cell death. (**a**) Cells were pretreated with z-VAD-fmk (20 *μ*M) for 2 h and then incubated with control or celastrol for 24 h. Cell viability was assessed by MTS. (**b**) The levels of AIF and Endo G in the mitochondria and cytosol were determined by western blot in HOS cells. (**c**) Cells were treated with various concentrations of celastrol for 24 h or incubated with celastrol (3 *μ*M) for different hours. The level of LC3B was measured by western blot. (**d**) Cells treated with or without celastrol for 24 h were collected and stained with acridine orange. Representative images of acridine orange-stained cells captured by fluorescent microscopy ( × 400) were shown. Bar: 50 *μ*m. (**e**) Transmission electron microscopy was utilized to observe the formation of autophagosome and ultrastructural change of nucleus. Arrows indicate autophagosomes containing intact and degraded cellular debris. Asterisks indicate nuclear condensation. Bar: 1 *μ*m. (**f**) z-VAD-fmk (20 *μ*M), 3-MA (2.5 mM for HOS, 5 mM for MG-63) or combination of them was added to cells 2 h before celastrol treatment. After 24 h, cell viability was determined. *P<0.05 *versus* control, ^#^*P*<0.05 *versus* celastrol treatment

To determine whether apoptosis is responsible for the inhibition of cell growth induced by celastrol, we performed Hoechst 33258 staining, TEM and flow cytometry assay. Figures 2a and 3e show that celastrol-induced apoptotic chromatin condensation and DNA fragmentation were clearly observed. To quantify the apoptosis, cells treated with celastrol were stained with annexin V-PE/7-AAD. The proportion of apoptosis was negligible for control cells, whereas 24 h of exposure of cells to celastrol resulted in a dose-dependent increase of both early and late apoptotic cells (Figure 2b). Next, we investigated the effect of celastrol on mitochondria. Figure 2c reveals that mitochondrial membrane potential (MMP) sharply decreased following celastrol treatment. Overall, these results clearly indicate that celastrol induces mitochondria-mediated apoptosis.

### Celastrol induces caspase-dependent apoptosis through the extrinsic and intrinsic pathways

Apoptosis can be induced either by extrinsic stimuli through cell surface death receptors or by intrinsic stimuli through the mitochondrial signaling pathway.^30^ Thus, we attempted to determine which pathway was involved. As shown in Figure 2d, celastrol markedly activated caspase-3, -8 -9 and led to PARP cleavage. To confirm caspase results, we performed caspase activity assay. Figure 2f shows that caspase-3, -8 and -9 activities increased with escalating doses of celastrol. Then we investigated DR4, DR5, TRAIL, Fas and FasL proteins, major members of the extrinsic pathway. Figure 2e demonstrates that celastrol upregulated the expression of DR5, but had minimal effect on DR4, TRAIL, Fas or FasL (data not shown). Moreover, following celastrol treatment, Bid, the BH3-only pro-apoptotic Bcl-2 family member, was cleaved by active caspase-8 to truncated Bid (tBid), which translocated to mitochondria to trigger the intrinsic pathway (Figure 2e).^31^ To further confirm these findings, we investigated the roles of caspases using z-VAD-fmk, z-IETD-fmk and z-LEHD-fmk. As expected, we observed a moderate inhibitory role of either z-IETD-fmk or z-LEHD-fmk in the celastrol-induced apoptosis while z-VAD-fmk had a more potent inhibitory effect (Figure 2g). All the data imply that celastrol induces caspase-dependent apoptosis by activating both the extrinsic and intrinsic pathways.

### Celastrol triggers autophagy, which contributes to celastrol-induced cell death

To understand the role of apoptosis in the celastrol-induced cell death, we examined cell viability in the presence of z-VAD-fmk. Unexpectedly, we found that z-VAD-fmk only caused a partial reduction in the celastrol-induced cell death (Figure 3a), implying that other forms of cell death may be involved. Then we investigated the expressions of AIF and Endo G, two important factors that mediate apoptosis through the caspase-independent pathway.^32, 33^ Figure 3b shows that celastrol had minimal effect on the release of AIF or Endo G from mitochondria into cytosol. Next, we measured the autophagy marker protein LC3B to determine whether autophagy was induced. Figure 3c shows that celastrol increased the level of LC3B-II in HOS and MG-63 cells. We also observed that celastrol led to the accumulation of bright red acidic vesicles resembling autolysosomes (Figure 3d). TEM was used to directly demonstrate autophagosome formation. Figure 3e shows that, concurrent with apoptotic chromatin condensation, numerous large autophagic vacuoles in the cytoplasm were observed, in which the vacuolar contents were degraded, evidence for the impact of celastrol in the regulation of autophagic formation in osteosarcoma cells.

Autophagy could either promote cell survival or act as an alternative mechanism of programmed cell death.^34^ To clarify the role of autophagy, cell viability in the presence of 3-MA, the autophagy inhibitor, was assessed. We also analyzed cell viability in response to the combination of z-VAD-fmk and 3-MA to confirm the coactivation of these two cell death forms. 3-MA moderately diminished celastrol-induced cell death by ~10% (Figure 3f). Interestingly, combination of z-VAD-fmk and 3-MA potently abolished the cell death.

These data reveal that autophagy induced by celastrol serves a pro-death function, and celastrol triggers both apoptosis and autophagic cell death in osteosarcoma cells.

### Celastrol induces JNK activation, which is required in celastrol-induced apoptosis

**Figure 4:**
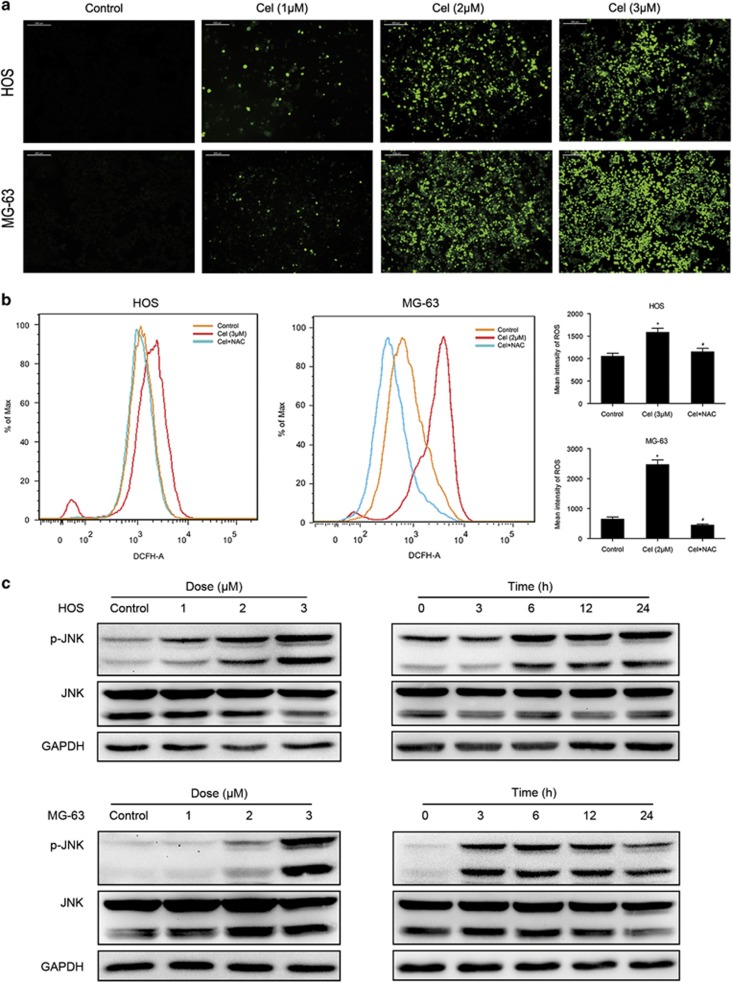
ROS generation and JNK activation are triggered by celastrol. (**a**, **b**) Cells were treated with celastrol for 12 h and then loaded with DCFH-DA for 30 min. The level of ROS was determined by fluorescence microscopy ( × 200) and flow cytometry. Representative images were presented. Quantitative analysis of ROS generation was shown in histograms. Bar: 200 *μ*m. *P<0.05 *versus* control, ^#^*P*<0.05 *versus* celastrol treatment. (**c**) Cells were treated with various concentrations of celastrol for 24 h or incubated with celastrol (3 *μ*M) for different hours. Levels of phospho-JNK and total JNK were determined by western blot

**Figure 5:**
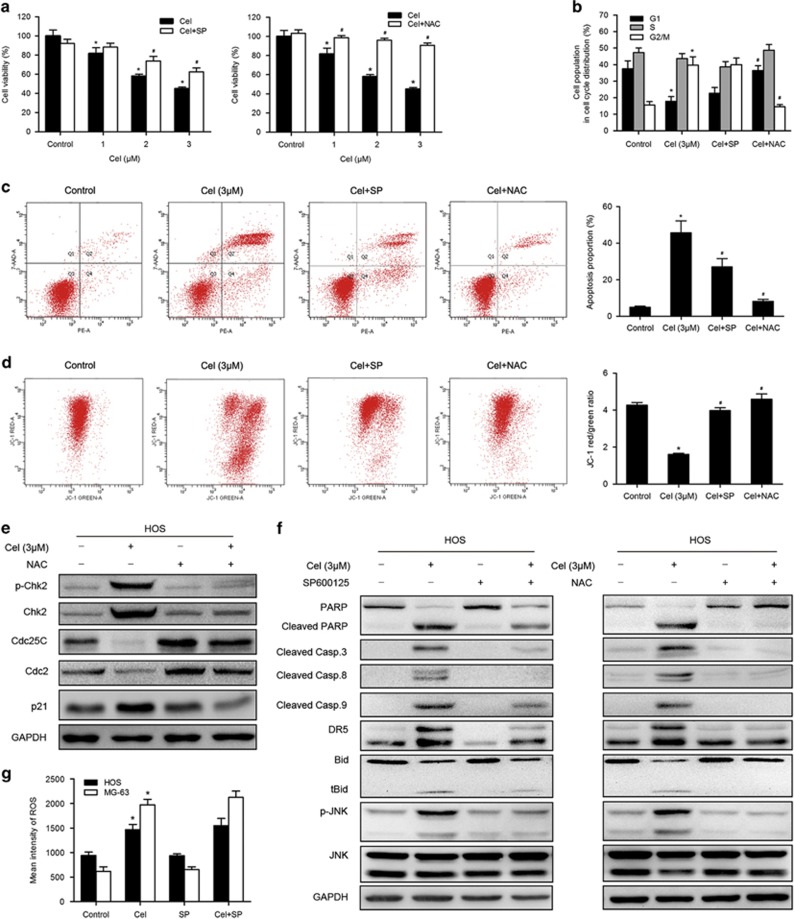
Roles of ROS and JNK in G2/M phase arrest and apoptosis induced by celastrol. HOS cells were preincubated with SP600125 (40 *μ*M) or NAC (5 mM) for 1 h, and then treated with celastrol (3 *μ*M) for 24 h. (**a**) Cell viability was measured by MTS. (**b**) Cell cycle was evaluated by flow cytometry. The percentage of cell cycle distribution was presented in histograms. (**c**, **d**) Induction of apoptosis and changes of mitochondrial membrane potential were assessed by flow cytometry. Quantitative analysis in histograms was presented. (**e**) The expressions of cell cycle-regulated proteins were measured by western blot. (**f**) Changes of apoptosis-related proteins. phospho-JNK and total JNK were measured by western blot. (**g**) The level of ROS was determined by flow cytometry. Quantitative analysis of ROS generation was shown in histograms. *P<0.05 *versus* control, ^#^*P*<0.05 *versus* celastrol treatment

We investigated the effect of celastrol on JNK activation. Figure 4c shows that celastrol increased the level of JNK phosphorylation in both HOS and MG-63 cells. To determine the contribution of activated JNK to celastrol-induced apoptosis or cell cycle arrest, we used the specific JNK inhibitor, SP600125 (SP). MTS assay showed that SP could effectively reduce the cell death caused by celastrol (Figure 5a). Flow cytometry assay indicated that SP attenuated the celastrol-induced apoptosis and inhibited depolarization of mitochondria (Figures 5c and d). Western blot analysis showed that SP inhibited JNK phosphorylation and activation of apoptosis-related proteins to a great extent (Figure 5f). However, SP failed to restore the celastrol-induced increase in the G2/M population (Figure 5b). These results suggest that the activation of JNK is required for celastrol-induced apoptosis but not involved in G2/M phase arrest.

### Celastrol induces ROS generation, which is the proximal event of JNK and acts as an initiator in celastrol-induced apoptosis and G2/M phase arrest

ROS have been shown to participate in the regulation of apoptosis and cell cycle arrest,^35, 36^ and also promote the sustained JNK activation.^26, 37^ As shown in Figure 4a, ROS generation was initiated by 1 *μ*M celastrol and explosively increased by 2, 3 *μ*M celastrol at 12 h. To further confirm the role of ROS in celastrol-induced cell death, NAC, ROS scavenger, was used. Figure 4b shows that NAC blocked the increased ROS induced by celastrol. MTS assay showed that NAC reversed the cell death (Figure 5a). In contrast to the effect of JNK inhibitor, NAC much more potently abolished the apoptosis and the decrement of MMP (Figures 5c and d). Western blot analysis demonstrated that NAC completely inhibited celastrol-induced activation of apoptosis-related proteins (Figure 5f). NAC also had a hard inhibitory effect on celastrol-induced G2/M phase arrest by reversing the key molecules of G2/M cell cycle regulator markers (Figures 5b and e). Furthermore, NAC strongly blocked JNK phosphorylation while the JNK inhibitor did not suppress but even slightly elevated ROS generation (Figures 5f and g). All these results reveal that ROS is the proximal event of JNK and most likely acts as an initiator in celastrol-induced apoptosis and G2/M phase arrest.

### Autophagy is mediated by JNK activation and ROS generation

**Figure 6:**
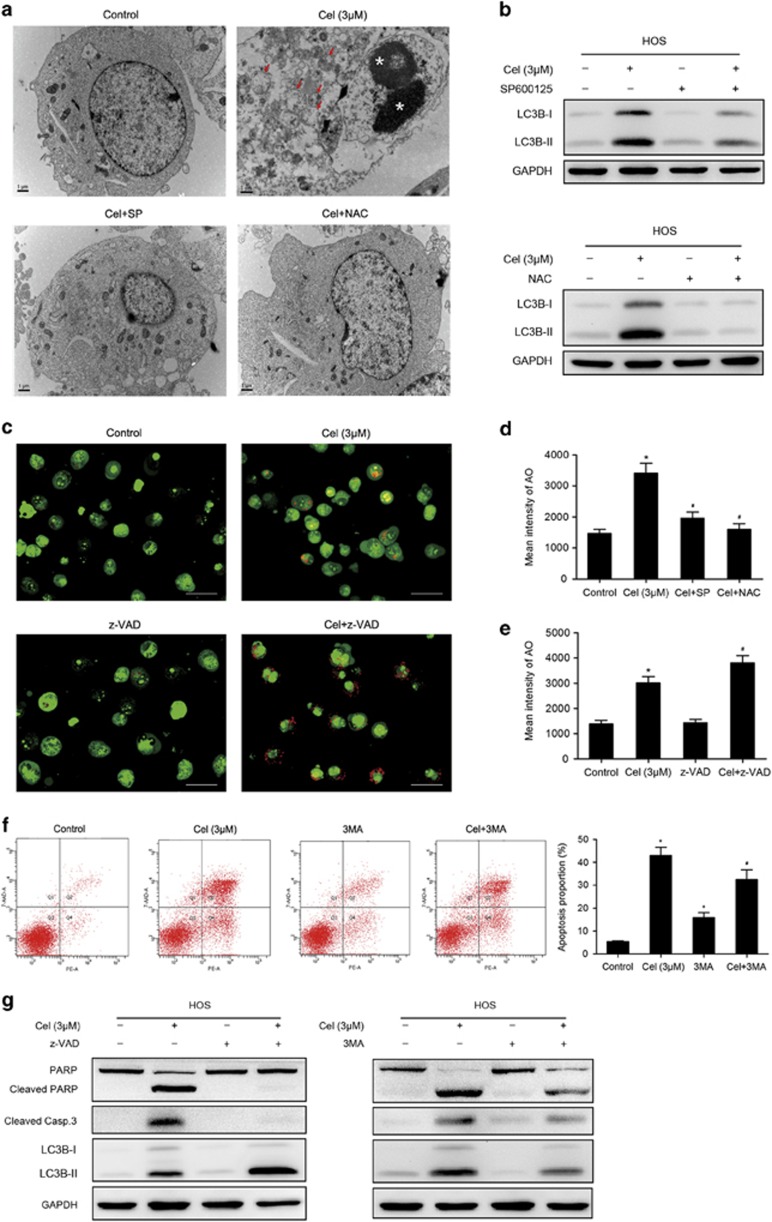
Roles of ROS and JNK in autophagy and the interplay between apoptosis and autophagy. HOS cells were preincubated with SP600125 (40 *μ*M), NAC (5 mM) for 1 h, or 3MA (2.5 mM), z-VAD-fmk (20 *μ*M) for 2 h, and then treated with celastrol (3 *μ*M) for 24 h. (**a**) Transmission electron microscopy was utilized to evaluate the changes of autophagosome and nucleus. Arrows indicate autophagosomes and asterisks indicate nuclear condensation. Bar: 1 *μ*m. (**b**) The expression of LC3B was analyzed by western blot. (**c**–**e**) The level of acridine orange staining was determined by fluorescence microscopy ( × 400) and flow cytometry. Representative images were presented. Quantitative analysis of red fluorescence representing acidic vesicles was shown in histograms. Bar: 50 *μ*m. (**f**) Cells were stained with annexin V-PE/7-AAD and analyzed by flow cytometry. The chart illustrates apoptosis proportion from three separate experiments. (**g**) Levels of LC3B, cleaved PARP and caspase-3 were assessed by western blot. *P<0.05 *versus* control, ^#^*P*<0.05 *versus* celastrol treatment

To determine the importance of JNK activation and ROS generation in celastrol-induced autophagy, we analyzed the level of LC3B-II in the presence of SP or NAC. As shown in Figure 6b, both SP and NAC significantly suppressed the increased expression of LC3B-II induced by celastrol. Large autophagic vacuoles were scarcely observed in cells pretreated with SP or NAC (Figure 6a). The quantitative analysis of AO-stained cells revealed that SP and NAC potently diminished the intensity of red fluorescence (Figure 6d). These results suggest that autophagy triggered by celastrol is dependent on JNK activation and ROS generation.

### Inhibition of apoptosis enhances autophagy while suppression of autophagy diminishes apoptosis

Overwhelming evidence has elucidated the complex relationship between apoptosis and autophagy.^20^ We subsequently investigated their interplay. First, the inhibition of apoptosis on autophagy was determined. Figures 6c and e show that z-VAD-fmk increased accumulation of AO-staining red acidic vesicles induced by celastrol. Western blot assay demonstrated that inhibition of apoptosis enhanced the expression of LC3B-II (Figure 6g). This suggests that part of apoptotic cell death may have shifted to autophagic cell death when apoptosis was inhibited. Next, we examined the inhibition of autophagy on apoptosis. Celastrol-induced apoptosis was moderately blocked by 3MA, despite slight apoptosis was observed in HOS cells treated with 3MA alone (Figure 6f). Figure 6g shows that 3MA diminished cleavage of caspase-3 and PARP to a certain extent. These results suggest that inhibition of apoptosis enhances autophagy while autophagy might contribute to apoptosis.

### Celastrol has the similar effects on primary osteosarcoma cells

We obtained primary cells derived from nine patients suffered from osteosarcoma to verify whether celastrol has the similar effects on clinical specimens. As expected, celastrol inhibited the proliferation of all primary cells. Supplementary Table S1 demonstrates the IC_50_ values of celastrol and the information of specimens. We chose two types of cells, namely OS-718 and OS-1227, to determine whether celastrol induces apoptosis, autophagy, ROS and JNK activation. As expected, celastrol treatment activated caspase-3, -8, -9, DR5 and cleavage of PARP, Bid, upregulated the expression of LC3B-II, increased JNK phosphorylation and ROS generation dose dependently (Supplementary Figures S1A–C). These data reveal that celastrol has the similar effects on primary osteosarcoma cells.

### Celastrol inhibits growth of osteosarcoma *in vivo*

**Figure 7:**
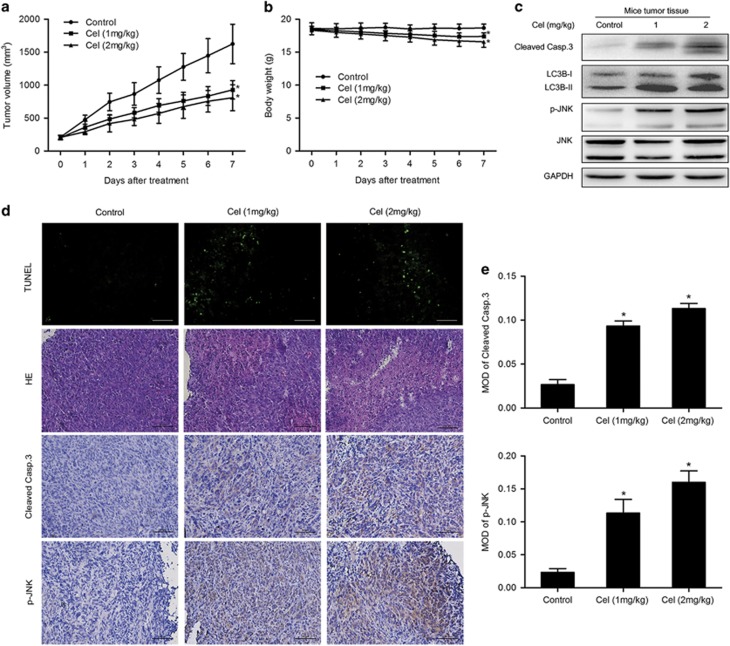
Celastrol inhibits growth of human osteosarcoma xenograft *in vivo*. HOS cells were inoculated subcutaneously in the right flank of BALB/c-nu mice. Intraperitoneal administration of vehicle or celastrol (1 or 2 mg/kg) daily was started when tumor volume reached around 200 mm^3^. When the tumors of control group reached around 1600 mm^3^, all mice were killed. (**a**, **b**) Tumor sizes and body weights were measured daily. (**c**) The levels of cleaved caspase-3, phospho-JNK and total JNK in tumor xenograft tissues were measured by western blot. (**d**) The apoptotic status of tumor tissues was assessed by TUNEL assay. H&E staining was used to evaluate the histology. The expression levels of cleaved caspase-3 and phospho-JNK were also examined by immunohistochemistry. Representative images were presented. Bar: 50 *μ*m. (**e**) Mean optical density of cleaved caspase-3 and phospho-JNK was quantified by Image Pro-Plus. *P<0.05, significantly different compared with control

*In vivo* effect of celastrol on osteosarcoma was determined via intraperitoneal administration in a tumor-transplanted mouse model. Celastrol at doses of 1 and 2 mg/kg resulted in significant decrease in tumor volume by 42.9 and 50.2%, respectively, after 7 days of drug administration (Figure 7a). It is worth noting that 1 and 2 mg/kg celastrol treatment caused 5.7 and 9% of weight loss in mice, respectively (Figure 7b). H&E staining and TUNEL assay demonstrated more dead cells and the evident increase in apoptosis proportion in celastrol-treated tumor tissues (Figure 7d). Figure 7c shows that celastrol increased levels of cleaved caspase-3, LC3B-II and phospho-JNK. Immunohistochemistry demonstrated the increase in mean areas that stained positively for cleaved caspase-3 and phospho-JNK in celastrol-treated tumor tissues, which was quantified by IPP software in terms of mean optical density (MOD) (Figures 7d and e). All the results reveal that celastrol inhibits growth of osteosarcoma *in vivo* with low levels of toxicity.

## Discussion

Despite the prognosis of localized osteosarcoma has markedly improved due to new therapeutic developments, the long-term survival has stagnated in the past several decades. Innovative drugs are needed for further improvement of outcome in osteosarcoma patients. Many reports have highlighted that celastrol is becoming an effective, safe and desirable approach to the treatment of cancers.^6, 7, 8, 9, 10, 11, 12^ Our results presented here confirm that celastrol could inhibit proliferation of human osteosarcoma *in vitro* and *in vivo* through G2/M arrest, apoptosis and autophagy mediated by the ROS/JNK signaling pathway.

Anticancer insights derived from cell cycle research have given birth to the idea of cell cycle G2 checkpoint abrogation as a cancer-cell-specific therapy.^38^ The cyclin B1/Cdc2 complex, which is kept inactive by phosphorylation of Cdc2, has a key role in promoting the G2/M phase transition.^39^ At the onset of mitosis, Cdc25C, a dual specificity phosphatase, is activated for dephosphorylation of Cdc2. The checkpoint kinases Chk1 and Chk2 phosphorylate Cdc25C, which downregulates Cdc25C activity.^14, 15^ Our studies showed that celastrol caused G2/M phase arrest through upregulation of phospho-Chk2, Chk2, phospho-Cdc25C, phospho-Cdc2, p21 and downregulation of Cdc2, Chk2. However, surprisingly, celastrol increased the level of cyclin B1. Similar results had been reported before.^40, 41^ This may be explained by the fact that suppression of Cdc2 activity could prevent cyclin B1 degradation by ubiquitin-dependent proteolysis, which led to the increase of cyclin B1.^41^ In addition, p21 has an important role in G2/M checkpoint through inhibition of the Cdc2/cyclin B1 complex in a p53-dependent or independent manner.^16, 42^ As HOS is p53-mutant, it is most likely that upregulation of p21 was mediated in a p53-independent manner and the specific mechanism needs to be further explored.

Apoptosis is a major route to eradicate cancers. Here, we revealed that celastrol induced apoptosis by activating both extrinsic and intrinsic pathways. Surprisingly, the caspase inhibitor could not entirely prevent the cell death, leading us to other caspase-independent pathways. Lee *et al.*^12^ and Yang *et al.*^43^ found that AIF played a critical role in celastrol-induced apoptosis independent of caspases. Besides that, Endo G, another apoptogenic protein in the intermembrane space of mitochondria, can translocate to the nucleus and directly digest nuclear DNA in the absence of caspase activity.^33^ Accordingly, we examined the expressions of these two proteins. However, no detectable change of AIF or Endo-G from mitochondria to cytosol was observed in celastrol-treated cells.

Autophagy, another caspase-independent cell death pathway, was investigated. We found that autophagy was induced as evidenced by accumulation of AO-staining acidic vesicles, formation of autophagosomes observed with TEM and upregulation of LC3B-II. We also revealed that the celastrol-induced cell death, moderately diminished by 3-MA, was markedly restored by combination of 3-MA and z-VAD-fmk, indicating that celastrol induced cell death through both apoptosis and autophagy. Several studies reported that autophagy served as a kind of survival mechanism in celastrol-treated cancers.^44, 45, 46^ In contrast, our study revealed that autophagy induced by celastrol contributed to the cell death.

Considerable evidence has delineated the complex interplay between apoptosis and autophagy, which can cooperate, antagonize or assist each other.^20, 47^ In our study, z-VAD-fmk increased accumulation of AO-staining acidic vesicles and the expression of LC3B-II, indicating that, when apoptosis was blocked, cells preferentially died through an autophagic pathway. In contrast, suppression of autophagy diminished cleavage of caspase-3 and PARP, indicating that autophagy contributed to apoptosis. Further work is needed to clarify the molecular connections between apoptosis and autophagy.

Next, we explored the upstream pathways. It is well-documented that excessive generation of ROS could interfere with cellular signaling pathways,^22, 23, 24^ and JNK also has a pivotal role in many cellular events.^28, 29, 48^ In the present study, celastrol induced a significant increase in ROS generation and JNK phosphorylation. The ROS inhibitor, NAC, completely reversed the celastrol-induced inhibition of cell proliferation, apoptosis and autophagy, while the JNK inhibitor significantly attenuated them. Moreover, NAC strongly blocked the G2/M phase arrest but the JNK inhibitor failed to, indicating that ROS but not JNK modulated the celastrol-induced cell cycle arrest. In addition, JNK phosphorylation was potently abolished by NAC but ROS generation was not attenuated by the JNK inhibitor, implying that ROS is the proximal event of JNK. From these data, we concluded that celastrol induced apoptosis and autophagy through the ROS/JNK signaling pathway and ROS had a vital role in G2/M phase arrest caused by celastrol.

Interestingly, previous studies have reported that celastrol has antioxidant properties on microglia and endothelial cells, also attenuates hypertension-induced oxidative stress in vascular smooth muscle cells (VSMCs).^4, 49^ These findings conflict with our results that celastrol induces ROS generation in osteosarcoma cells. On one hand, this discrepancy may be attributed to the difference between non-cancerous cells (microglia, endothelial cells and VSMCs) and cancer cells (osteosarcoma). On the other hand, dose probably matters. Celastrol suppresses oxidative stress at nanomolar concentrations (10–100 nmol/l). However, in our study, the concentration of celastrol inducing detectable ROS is no less than 1 *μ*M, consistent with the dose of celastrol in other cancers.^12^ Taken together, the effect of celastrol on oxidative stress is most likely dependent on cell type and concentration.

Yang *et al.*^43^ demonstrated that treatment of prostate tumor-bearing nude mice with celastrol resulted in significant inhibition (65–93%) of the tumor growth without overall gross toxicity.^9^ In our study *in vivo*, we found that celastrol at doses of 1 and 2 mg/kg significantly inhibited tumor growth (42.9–50.2%). Western blot and immunohistochemical analysis confirmed the increase in cleaved caspase-3, LC3B-II and phospho-JNK following celastrol treatment. It is noteworthy that celastrol caused 5.7–9% of weight loss in mice, revealing that high dose of celastrol may have a few side effects to some extent.

**Figure 8:**
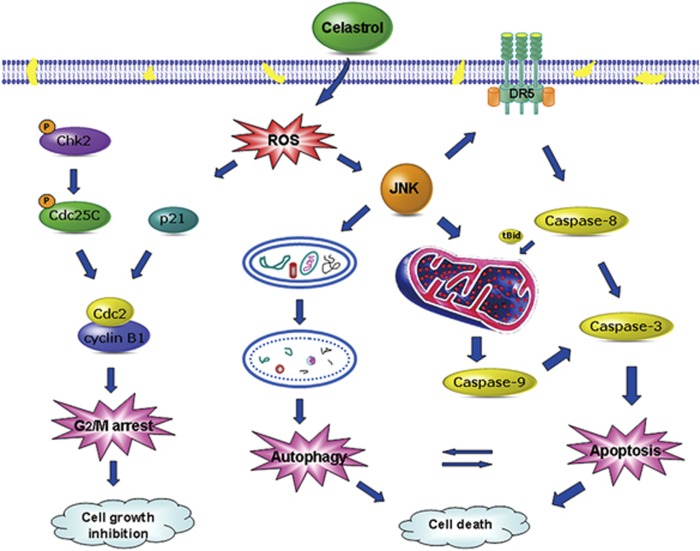
A schematic diagram of the pathways in which celastrol induces cell growth inhibition and cell death in osteosarcoma

In conclusion, our study is the first to demonstrate that celastrol can effectively inhibit the proliferation of osteosarcoma cells by causing G2/M phase arrest, and lead to cell death by inducing apoptosis and autophagy mediated by the ROS/JNK signaling pathway (Figure 8). In the osteosarcoma xenograft model, celastrol also shows significant antitumor activity with low levels of toxicity. This compelling evidence expands our understanding of the benefits and clinical application of celastrol therapy.

## Materials and methods

### Reagents and antibodies

Celastrol with purity greater than 98%, *N*-Acetyl-L-cysteine (NAC), SP600125, 3-Methyladenine (3-MA) were purchased from Sigma-Aldrich (St. Louis, MO, USA). Dulbecco's Modified Eagle Medium (DMEM), RPMI 1640 Medium, fetal bovine serum (FBS), penicillin, streptomycin, PBS and 0.25% trypsin were purchased from Gibco/BRL (Gaithersburg, MD, USA). The broad-spectrum caspase inhibitor (z-VAD-fmk) was obtained from Millipore (Billerica, MA, USA). Caspase-8 specific inhibitor (z-IETD-fmk) and caspase-9 specific inhibitor (z-LEHD-fmk) were purchased from BioVision (Mountain View, CA, USA). Antibodies against TRAIL and DR4 were obtained from ProteinTech Group (Chicago, IL, USA). Antibodies against caspase-3, caspase-8, caspase-9, poly (ADPribose) polymerase (PARP), DR5, Bid, Fas, FasL, phospho-JNK, JNK, LC3B, phospho-Cdc2, Cdc2, phospho-Cdc25C, Cdc25C, cyclin B1, phospho-Chk2, Chk2, p21, AIF, Endo G and GAPDH were purchased from Cell Signaling Technology (Beverly, MA, USA).

### Cell and cell culture

The human osteosarcoma cell lines HOS (CRL-1547TM, ATCC), MG-63 (CRL-1427TM, ATCC), U-2OS (HTB-96TM, ATCC), Saos-2 (HTB-85TM, ATCC) were from Cell Bank of Shanghai Institute of Biochemistry and Cell Biology, Chinese Academy of Sciences (Shanghai, China). OS-718 and OS-1227 are primary osteosarcoma cells derived from patients. FB-1,-2 and -3 are human primary skin fibroblasts, the gift from Dr Zheng Min. HOS, MG-63, Saos-2 and primary cells were cultured in DMEM, U-2OS in RPMI 1640, supplemented with 10% FBS, 100 U/ml penicillin and 100 *μ*g/ml streptomycin. Cells were maintained at 37 °C in a humidified incubator of 5% CO_2_.

All procedures involved clinical specimens were approved by the Human Research Ethics Committee of the Second Affiliated Hospital of Zhejiang University School of Medicine, China.

### Cell viability assay

The anti-proliferative effect of celastrol on osteosarcoma cells was determined by the MTS kit (cellTiter96AQ, Promega, Madison, WI, USA). Briefly, cells were seeded in 96-well plates with a density of 3–7 × 10^3^ cells/well. After 12 h, they were treated with various concentrations of celastrol (0–8 *μ*M) for different periods of time (0–48 h). The fresh mixture of MTS and PMS was added and incubated for 2–4 h at 37 °C following the manufacturer's protocol. A MR7000 microplate reader (Dynatech, NV, USA) was used to measure the absorbance at 490 nm, and IC_50_ values were calculated using the probit model. Data represented the mean of five replicates. Each performed in triplicate.

### Clone formation assay

Cells were seeded at 100 cells/well in six-well plates and dispersed evenly by shaking the dishes slightly. After attachment, cells were treated with celastrol at varying concentrations (0–2 *μ*M) for about 14 days when the cells grew to visible colonies. The medium was discarded and the cells were washed with PBS twice. After being fixed with 4% paraformaldehyde for 20 min, washed with PBS, the cells were stained with 0.1% crystal violet for 15 min. Finally, the dye was washed with PBS. The clones with more than 50 cells were counted under an ordinary optical microscope.

### Morphological apoptosis

Characteristic apoptotic morphological changes were assessed by fluorescent microscopy using Hoechst 33258 staining. In brief, cells were exposed to celastrol for 24 h and then stained with Hoechst 33258 for 10 min. After being washed twice with PBS, cells were observed with a fluorescence microscope (Olympus, Tokyo, Japan) to determine nuclei fragmentation and chromatin condensation.

### Transmission electron microscopy observation

Changes in cell ultra-structure caused by celastrol were visualized using transmission electron microscopy (TEM). Apoptosis was assessed by observation of nuclear condensation and autophagy was evaluated by examining autophagosome formation. The treated cells were fixed with 2.5% glutaraldehyde and post-fixed with 1% osmium tetroxide. After being dehydrated in increasing concentrations of alcohol, the cell pellets were embedded in epon. Representative areas were chosen for ultrathin sectioning and examined on a transmission electron microscope at a magnification of × 10 000.

### Apoptosis analysis

Apoptosis was determined by Annexin V-PE/7-AAD kit (BD Biosciences, San Diego, CA, USA). In brief, cells were seeded in six-well plates with a density of 2 × 10^5^ cells/well and treated with celastrol at concentrations ranging from 0 to 3 *μ*M for 24 h. The cells were incubated with PE-conjugated annexin V and 7-AAD for 15 min at room temperature in the dark. Then cells were washed with ice-cold PBS, resuspended with 300 *μ*l PBS. Samples were analyzed by flow cytometer (FACSCalibur, BD, San Jose, CA, USA) and CELLQuest software (FACSCalibur).

### Analysis of caspase-3, -8, -9 activities

Caspase activities were measured using Caspase Activity Kit (Beyotime biotechnologies, Jiangsu, China) according to the manufacturer's instructions. Lysates of cells were prepared after being treated with various concentrations of celastrol for 24 h. Activities of caspase-3, -8 and -9 were measured by substrate peptides Ac-DEVD-pNA, Ac-IETD-pNA and Ac-LEHD-pNA, respectively. The release of pNA was quantified by measuring the absorbance with the MR7000 microplate reader at 405 nm. Caspase activity was expressed as the ratio of treated cells to the control.

### Measurement of mitochondrial membrane potential

The mitochondrial membrane potential (MMP) was measured with JC-1 fluorescent probe (Beyotime). In brief, 2 × 10^5^ cells were exposed to celastrol (0–3 *μ*M) for 24 h. Collected cells were incubated with JC-1 for 20 min at 37 °C. The stained cells were washed twice and analyzed by a flow cytometer. Mitochondrial depolarization was indicated by a decrease in the red/green fluorescence intensity ratio.

### Measurement of ROS

Generation of intracellular ROS was measured by a ROS Assay with DCFH-DA (Beyotime). In brief, cells were plated at a density of 2 × 10^5^ cells/well in six-well plates and exposed to celastrol (0–3 *μ*M) for 12 h. Then the cells were incubated with DCFH-DA (10 *μ*M) for 30 min at 37 °C. The level of ROS was determined by fluorescence microscopy and flow cytometer.

### Cell cycle analysis

Cell cycle was monitored by flow cytometry with PI/RNase staining buffer (BD Biosciences). In brief, 5 × 10^5^ cells were exposed to celastrol and fixed with 70% ethanol at −20 °C overnight. Then the cells were stained with PI/RNase staining buffer for 15 min and analyzed by flow cytometer and ModFit LT software (FACSCalibur). For each measurement, 2 × 10^4^ cells were analyzed and the representative measurements were shown.

### Detection of acidic vesicular organelle

To detect the formation of acidic vesicular organelle (AVO), cells were stained with acridine orange (AO) (Sigma-Aldrich). In the stained cells, the cytoplasm and nucleus fluoresce bright green, while the acidic vesicular organelles fluoresce bright red. In brief, 2 × 10^5^ cells were exposed to celastrol for 24 h. After being rinsed with fresh medium, the cells were incubated with AO (1 *μ*g/ml) for 15 min at 37 °C. Cells were washed three times with PBS and then observed under fluorescence microscopy. To quantify the development of AVO, the stained cells were also analyzed by flow cytometer.

### Western blot analysis

Cells were seeded at a density of 5 × 10^5^ cells in 60-mm dishes and treated with celastrol for 24 h, followed by centrifugation. The pellets were lysed in RIPA lysis buffer containing protease inhibitor cocktail (Sigma-Aldrich) for 30 min on ice. Cell lysates were centrifuged at 13 000 g for 15 min and the supernatant was collected. To examine the subcellular location of AIF, cytosolic extracts were prepared according to the manual provided in the mitochondria isolation kit (Pierce, Waltham, MA, USA). Protein content was quantified using a BCA protein assay kit (Pierce) according to the manufacturer's instruction. Equal amounts of protein (40 *μ*g) were separated by electrophoresis on 10–12% SDS-PAGE at 100 V for 1.5 h. Then the proteins were transferred onto polyvinylidene difluoride (PVDF) membrane (Millipore) at 250 mA for 2 h. The membranes were blocked with 5% bovine serum albumin (Sigma-Aldrich) for 1 h at room temperature and then incubated with primary antibody at 4 °C overnight. After being washed five times with TBST, the membranes were incubated with an HRP-conjugated secondary antibody for 1 h at room temperature. Each band was visualized by enhanced chemiluminescence kit (Millipore).

### Human osteosarcoma xenograft experiment

Female BALB/c-nu mice, aged 4 weeks, were purchased from Shanghai Laboratory Animal Center of Chinese Academy of Sciences. They were maintained under specific pathogen-free conditions and supplied with sterilized food and water. On day 0, 5 × 10^6^ HOS cells suspended in 0.1 ml PBS were inoculated subcutaneously in the right flank of each mouse (six mice each group). On day 9, when the tumors reached palpable size of around 200 mm^3^, mice were randomly assigned to three groups and received daily intraperitoneal injection with 100 *μ*l of vehicle (10% DMSO, 70% Cremophor/ethanol (3 : 1), and 20% PBS), and 1 or 2 mg/kg of celastrol. Tumor sizes were measured daily to observe dynamic changes in tumor growth and calculated by a standard formula: length × width^2^/2. Body weights were also measured daily. After 7 days of drug administration, when the tumors of control group reached around 1600 mm^3^, all mice were killed. Tumors were dissected and stored in liquid nitrogen or fixed in formalin for further analysis. All treatment protocols were approved by the Animal Care and Use Committee of Zhejiang University, China.

### TUNEL assay

The terminal deoxynucleotidyl transferase-mediated dUTP nick-end labeling (TUNEL) assay was used to evaluate the apoptotic response of tumor tissues with In Situ Cell Death Detection Kit, Fluorescein (Roche Diagnostics, Mannheim, Germany). In brief, after being deparaffinized and hydrated, slides were washed with PBS twice and incubated with proteinase K (20 *μ*g/ml) for 25 min at 37 °C. After a second round of washes, slides were incubated with TUNEL reaction mixture prepared freshly for 1 h at 37 °C in a moist chamber. After being washed twice, the slides were observed under fluorescence microscopy.

### Tumor histology

Formalin-fixed, paraffin-embedded tumor specimens were cut into serial sections (5 *μ*m). Following a hydration process, the slides were stained with hematoxylin for 15 min and immersed in 1% hydrochloric acid in 75% ethanol for 30 s. Before dehydration, the slides were stained with eosin for 5 min. Finally, the slides were immersed in xylene and mounted.

### Immunohistochemical analysis and quantification

For immunohistochemical staining, slides were deparaffinized in xylene and hydrated with graded alcohol, and treated with 3% H_2_O_2_ for 15 min to block endogenous peroxidase activity. Antigen retrieval was completed in boiling sodium citrate buffer (PH 6.0) for 10 min. Then slides were incubated with 10% normal goat serum for 15 min, followed by incubation with cleaved caspase 3 and phospho-JNK antibodies at 4 °C overnight in a moist chamber. After being washed three times with PBS, slides were incubated with the secondary antibody at 37 °C for 30 min. Immunoreactivity was visualized by incubation with DAB (Sigma-Aldrich). Hematoxylin was used for background counterstaining.

For quantification, three random × 400 microscopic fields per slide were photographed using a DP70 CCD camera (Olympus) coupled to an AX-70 microscope (Olympus). Image pro-plus 6.0 (IPP, Media Cybernetics, BD Biosciences) was used for digital photographs analysis referencing the method introduced by Xavier *et al.*^50^ The measure parameters included density mean, area sum and IOD. After the background staining correction, the optical density was calibrated and the area of interest was set through: hue, 0–30; saturation, 0–255; intensity, 0–230. Then the image was converted to gray scale image and values were counted.

### Statistical analysis

The quantitative data were shown as means±S.D. and the statistical differences were calculated by one-way ANOVA analysis of variance with Dunnett's test or unpaired Student's t-test. The Mann–Whitney *U-*test was used to examine the significance of cleaved caspase 3 and phospho-JNK expression determined by IPP.

All statistical analyses were performed using the SPSS software (version 16.0, SPSS, Chicago, IL, USA). Tests were two-tailed and statistical significance was defined as *P*<0.05.

## Abbreviations

PCD: programmed cell death
JNK: c-Jun N-terminal kinase
ROS: reactive oxygen species
DR5: death receptor 5
AVO: acidic vesicular organelle
AO: acridine orange
MMP: mitochondrial membrane potential
TEM: transmission electron microscopy
AIF: apoptosis inducing factor
Endo G: endonuclease G
