# Supplementary figures and images for: Celastrol induces apoptosis and autophagy via the ROS/JNK signaling pathway in human osteosarcoma cells: an in vitro and in vivo study
Source: Cell Death Dis. 2015 Jan 22;6(1):e1604–. doi: 10.1038/cddis.2014.543 (PMC4669742; doi:10.1038/cddis.2014.543)

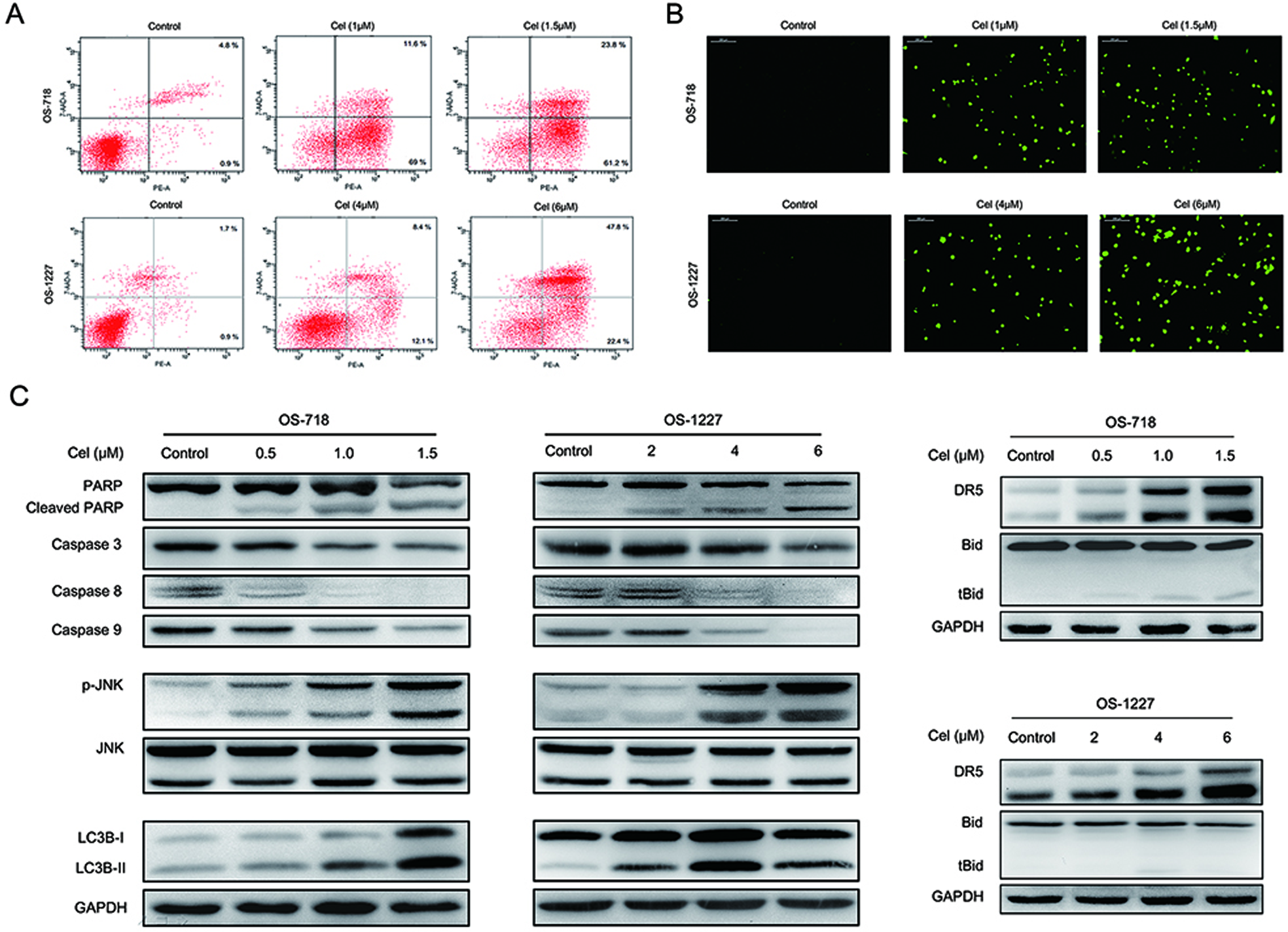

Supplement: Supplementary Figure S1 [file cddis2014543x2.tif]
